# Primary Hepatocytes Cultured on a Fiber-Embedded PDMS Chip to Study Drug Metabolism

**DOI:** 10.3390/polym9060215

**Published:** 2017-06-10

**Authors:** Yaowen Liu, Ke Hu, Yihao Wang

**Affiliations:** 1 College of Food Science, Sichuan Agricultural University, Yaan 625014, China; kehubeyond@163.com (K.H.); cdyihaow@163.com (Y.W.); 2 School of Materials Science and Engineering, Southwest Jiaotong University, Chengdu 610031, China

**Keywords:** fibers, microfluidic chips, hepatocytes, drug

## Abstract

In vitro drug screening using reliable and predictable liver models remains a challenge. The identification of an ideal biological substrate is essential to maintain hepatocyte functions during in vitro culture. Here, we developed a fiber-embedded polydimethylsiloxane (PDMS) chip to culture hepatocytes. Hepatocyte spheroids formed in this device were subjected to different flow rates, of which a flow rate of 50 μL/min provided the optimal microenvironment for spheroid formation, maintained significantly higher rates of albumin and urea synthesis, yielded higher CYP3A1 (cytochrome P450 3A1) and CYP2C11 (cytochrome P450 2C11) enzyme activities for metabolism, and demonstrated higher expression levels of liver-specific genes. In vitro metabolism tests on tolbutamide and testosterone by hepatocytes indicated predicted clearance rates of 1.98 ± 0.43 and 40.80 ± 10.13 mL/min/kg, respectively, which showed a good in vitro–in vivo correspondence. These results indicate that this system provides a strategy for the construction of functional engineered liver tissue that can be used to study drug metabolism.

## 1. Introduction

The liver is the major organ for drug biotransformation. In vitro drug screening is the primary alternative to animal testing due to fast, cheap, and safe drug development, with minimalized testing on animals. Numerous in vitro models have been proposed, such as liver slices, primary cells, and liver cell lines; however, they all have various disadvantages. Liver slices exhibit a limited lifespan in culture (approximately 1 week), limiting their widespread use in vitro [1]. Liver cell lines, such as HepG2, have no or very low levels of important drug-metabolizing enzymes and transporters [2]. Primary hepatocytes are currently the best option for drug testing; however, drug-metabolizing enzymes are rapidly altered after their isolation [3]. To improve the predictive abilities of primary hepatocytes, novel strategies need to be developed to maintain their morphology and function in vitro.

The ideal in vitro liver model for drug screening demands high reproducibility, high throughput, stability, sensitivity, and automated processing to enable its use in clinical research. On the basis of previous research, primary hepatocytes under conventional two-dimensional (2D) culture rapidly lose differentiated functions. Three-dimensional (3D) culture models mimic the in vivo environment of the liver; primary hepatocytes form 3D cell spheroids and enable extensive cell–cell interaction, which is important to maintain the function of biotransformation enzymes and transporter activities. Recently, many techniques have been developed to improve the utility of hepatocytes for drug development, and the 3D cell spheroid model is receiving increasing attention to study the influence of the liver microenvironment and drug testing under more physiologically relevant conditions [4]. Typical methods for spheroid formation include hanging drops, cell culture on non-adherent surfaces, pattern culture, and rotary cell culture systems [5]. However, traditional spheroid formation methods usually produce spheroids of different sizes, which is unsuitable for drug metabolism studies. This is because the size heterogeneity of spheroids causes size-dependent resistance of spheroids, affecting the reliability of the information for drug testing.

Recently, primary hepatocytes cultured in microfluidic chips have proven to be more powerful and sustainable than other available methods for drug screening. To mimic the cellular environments in vivo, microfluidics-based cell-culture platforms can be easily fabricated [6]. Given that primary hepatocytes can be cultured in a normal incubator, a particular spheroid can be easily aggregated [7]. Owing to cell–cell interactions [8], oxygen and nutrient delivery, metabolite removal, and shear stress, the viability and metabolic activities of hepatocytes can be maintained and improved [3]. However, most microfluidic chips are low throughput [9] because they are not compatible with the industrial-scale multi-well plates used for high-throughput drug screening [10].

Various approaches to build microfluidics-based cell-culture platforms have been widely reported. Schuler et al. fabricated a cell-based analog chip system containing two multi-chambers to coculture hepatocytes and lung cells. They also investigated drug metabolite–cell interactions of the system in terms of dose dynamics, and the system demonstrated great potential for use in drug-testing platforms [11]. Sung et al. developed a 3D hydrogel microfluidic chip, and liver, tumor, and marrow cells were cultured in separate chambers connected by channels to mimic blood flow, which reproduced multi-organ interactions and represented the pharmacokinetic and pharmacodynamic profiles of a drug in humans [12]. Gebhardt et al. constructed multi-well plate perfusion culture systems separated by a permeable polycarbonate membrane, and hepatocytes and intestinal bacteria were cocultured to investigate hepatic metabolism, hepatotoxicity, and enzyme induction. They found that the in vitro systems could represent the in vivo conditions well enough to study drug metabolism and enzyme induction [13]. Although the above approaches can be used to characterize drug metabolism, they are limited by the use of non-porous membranes as the substratum [14], which cannot provide precise control over chemical and mechanical microenvironments for cells [15]. In addition, issues such as complicated manufacturing processes and large-scale structures need to be addressed, which impede the success of drug evaluation [16].

Here, we developed an electrospun nanofiber matrix integrated into a polydimethylsiloxane (PDMS)-based microfluidic chip supporting the dynamic 3D culture of primary rat hepatocytes. Perfusion microchannels may offer multifunctional benefits to hepatocytes, providing effective transport, exchange of cell culture medium, and helping hepatocytes to grow into spheroids for more than 15 days. The viability and functions of 3D hepatocyte spheroids under different systems were optimized. Moreover, enzyme activities, expression levels of liver-specific genes, and in vitro intrinsic clearance were investigated. The results showed that the flow- and fiber-based microfluidic chip system could achieve longer hepatocyte culture, a higher level of hepatocyte functions, and more accurate prediction of drug metabolism compared to the available methods.

## 2. Materials and Methods

### 2.1. Materials

Poly(ethylene glycol)-poly(dl-lactide) (PELA; *M*_w_ = 42.3 kDa, *M*_w_/*M*_n_ = 1.23) was synthesized in our laboratory as follows. dl-Lactide and poly(ethylene glycol) (PEG) were prepared by bulk ring-opening polymerization using stannous octoate as the initiator. To modulate the graft efficiency of lactose into fibers, 4-armed lactosylated poly(dl-lactide) (lac-PLA; *M*_w_ = 7.6 k*D*a, *M*_w_/*M*_n_ = 1.32) was obtained by the polymerization of pentaerythritol and dl-lactide as described previously [17]. Proton nuclear magnetic spectra (^1^H-NMR; Bruker Avance DPX 300, Faellanden, Switzerland) were employed to determine the structure of lac-PLA. lac-PLA was dissolved in DMSO-D6 with tetramethylsilane as the internal standard. dl-Lactide, PEG, and pentaerythritol were purchased from Chemical Regents Company of China (Shanghai, China). PDMS (Sylgard 184 Silicone kit) was obtained from Dow Corning (Midland, MI, USA). Dimethyl sulfoxide (DMSO) was procured from Sigma-Aldrich (St. Louis, MO, USA). Goat anti-rat albumin antibody was obtained from Abcam (Cambridge, UK), and mouse anti-goat IgG-FITC was purchased from Biosynthesis Biotechnology Co. Ltd. (Beijing, China). 7-Benzyloxy-4-trifluoromethylcoumarin (BFC) and 7-methoxy-4-trifluoromethylcoumarin (MFC) were acquired from Tianjin Heowns Medicine Co. Ltd. (Tianjin, China). Tolbutamide and testosterone were purchased from Dalian Meilun Medicine Group Co. Ltd. (Dalian, China). All other chemicals and solvents were of reagent grade or better and were obtained from Chengdu Kelong Reagent Co. (Chengdu, China), unless otherwise indicated.

### 2.2. Fabrication of the Fiber-Based Microfluidic Chip

To maintain the viabilities and functions of hepatocytes [18], we used a pattered fiber support as a substrate to integrate with a microfluidic chip. Patterned fibers with a width of 200 µm and a thickness of 100 µm not only provide a 3D scaffold for hepatocytes but also act as a fluid channel. The patterned fiber was obtained by a patterned collector, which contained patterned silver circuits on a glass template, as previously described [19]. Electrospun patterned PELA/lac-PLA fibers were prepared by a traditional electrospinning method. In brief, the PELA solution and lac-PLA (5/5, *w*/*w*) solution in chloroform were added into a 2-mL syringe [18] and attached with a metal capillary shaped for clinical use. A steady flow at 1 mL/h from the capillary outlet was controlled by a syringe pump (Zhejiang University Medical Instrument Company, Hangzhou, China). The electrospinning voltage was set as 20 kV by a high-voltage statitron (Tianjin High Voltage Power Supply Company, Tianjin, China). The distance between the patterned collector and the spinneret was maintained at 15 cm. We fabricated a PDMS microchannel using a conventional soft lithographic process as previously described [20]. To make sure the patterned fiber was aligned well with the PDMS channel, a stereomicroscope was used to help place the pattered fibers into the grooved area of the upper PDMS layer. After the upper and lower layer were bonded [21], a pump was applied to connect the inlet and outlet ports by Teflon tubing (Genetec AB, Västra Frölunda, Sweden) to control the flow rate.

### 2.3. Cell Culture

Male SD rats aged 5–8 weeks old (Sichuan Dashuo Biotech Inc., Chengdu, China) were anaesthetized. All experimental procedures and protocols were reviewed and approved by the Animal Care and Use Committee of Sichuan Agricultural University and were in accordance with the Guide for the Care and Use of Laboratory Animals. Primary hepatocytes were isolated from the livers by a two-step in situ collagenase perfusion method. The viability of hepatocytes was determined by the trypan blue exclusion assay, and a viability of >90% was used. Fluorescein diacetate (FDA) were purchased from Molecular Probes (Carlsbad, CA, USA). The microfluidic channel was sterilized with 75% ethanol for 30 min prior to use. Hepatocytes were seeded at a density of 5 × 10^6^ cells per well on the various substrata. After cells attached for 10 h under a static environment, DMEM with 10% (*v*/*v*) fetal bovine serum was perfused into the microchannel at various flow rates. The microfluidics-based cell-culture platform was then placed in an incubator with 5% CO_2_ at 37 °C for 15 days.

### 2.4. Characterization of Cultured Hepatocytes

The viabilities of hepatocytes on different substrata were evaluated by a lactate dehydrogenase (LDH) assay kit (Nanjing Jiancheng Bioengineering Institute, Nanjing, China). The leakage of LDH released into the medium was quantified using a plate reader (Elx-800; Bio-Tek Instrument Inc., Winooski, VT, USA). The intracellular albumin produced by hepatocytes was observed by an immunofluorescent staining method. The microfluidics-based cell culture was washed twice with 2 mL PBS and then incubated with 0.1% Triton X-100 in PBS for 24 h at 4 °C. After 3 rinses with PBS, goat anti-rat albumin antibodies diluted with PBS were added and incubated at 4 °C for 24 h. The samples were washed 5 times with PBS, and the samples were incubated with mouse anti-goat IgG-FITC (fluorescein isothiocyanate) at 37 °C for 30 min. The albumin produced by the hepatocytes was observed by confocal laser scanning microscopy (CLSM, Olympus FV1000S, Tokyo, Japan). Hepatocyte morphology was assessed using a scanning step in the Z-direction with a size of 5 μm. Hepatocyte spheroid sizes were evaluated and processed by the Image-Pro 6.0 software (Media Cybernetics Inc., Bethesda, MD, USA) as described previously [17]. On days 1, 5, 10, and 15, the medium was collected and filtered. Albumin secretion and urea synthesis were measured with commercially available kits (Jiancheng Bioengineering Institute, Nanjing, China) according to the operation guide. The albumin and urea levels were normalized to the cell numbers per day (μg/10^6^ cells/day). The biliary excretion of hepatocytes was determined by FDA staining as described previously [18].

### 2.5. Characterization of Enzyme Activities of Cultured Hepatocytes

BFC and MFC were used as fluorescent substrates to measure the activities of CYP3A1 and CYP2C11 enzymes under different culture systems. Briefly, 100 μM BFC and 150 μM MFC were prepared with DMSO and culture media. After hepatocytes were cultured on different substrata in 96-well tissue culture plates (TCPs) for 1, 3, 5, 7, 10, and 15 days, different samples were treated with BFC and MFC during a 2 h incubation. Next, 20 μL of the metabolic solutions was collected, and 200 μL acetonitrile was added to stop the metabolic reactions. Samples were centrifuged, and supernatants were collected. The fluorescence intensities of BFC and MFC in the metabolites were measured using a fluorospectrophotometer (Hitachi F-7000, Tokyo, Japan). Standard curves of BFC and MFC were established with different concentrations of fluorescent substrates, and the enzyme activities were normalized to 10^6^ cells.

### 2.6. Gene Expression of Enzymes from Cultured Hepatocytes

Changes in gene expression of CYP3A1 and CYP2C11 enzymes were investigated by quantitative reverse-transcription polymerase chain reaction (RT-PCR) as described previously [22]. The sequences of primers for CYP3A1, CYP2C11, and β-actin are shown in Table 1. β-Actin was used as a housekeeping control. The cells were homogenized in Trizol reagent (Sigma-Aldrich). Total RNA was extracted using an RNeasy Plus Mini Kit (Qiagen Inc., Valencia, CA, USA), and the total RNA concentration was determined by optical densities at 260 nm using a spectrophotometer (BioTek Instrument Inc., Winooski, VT, USA) [23]. Then, RT-PCR was performed according to the manufacturer’s instructions. Briefly, cDNA was prepared from the total RNA using the High-Capacity RNA-to-cDNA Kit (Applied Biosystems Asia, Singapore) according to the manufacturer’s instructions. cDNA (10 ng) and 1 μL of 10 μM primer were added to 10 μL of the reaction mixture containing SYBR Green I master mix in a 96-well reaction plate. The thermocycling conditions were as follows: 95 °C for 10 s, 40 cycles of 95 °C for 5 s, and 60 °C for 20 s, followed by a dissociation curve step. The reaction was performed using a 7500 Fast Real-Time PCR system (Applied Biosystems Asia, Singapore), and gene transcription was evaluated using the ∆∆*C*t method. The expression level of each gene relative to that of β-actin was calculated [24].

### 2.7. Drug Metabolism Testing in Cultured Hepatocytes

To investigate the specific action of different drugs on hepatic-metabolism enzymes, testosterone and tolbutamide were chosen to study the hepatic clearance rates by CYP3A1 and CYP2C11, respectively [25]. Briefly, the drug solutions were dissolved in DMSO and diluted with cell culture media to a final concentration of less than 0.2% (*v*/*v*). After hepatocytes were cultured on different substrata for 7 and 15 days, different drug media were treated for 10, 20, 40, 60, and 120 min separately, after which 1.5 mL of the metabolic solutions were collected, and acetonitrile was added to stop the metabolic reactions. The amount of each drug was monitored by high-performance liquid chromatography (Agilent 1260 Infinity, Santa Clara, CA, USA) using a C18 column and an ultraviolet detector. Three independent experiments were set for each drug metabolism test to obtain statistically reliable data.

To determine the reusability of this model for drug testing, the intrinsic clearance of each drug cultured in different models for 7 days and 15 days was calculated according to the following formula:

CL_in vitro_ = (*C*_0_ − *C*_t_) × *V* × SF/AUC_0-t_ × *N* (1)

where *C*_0_ and *C*_t_ represent the concentrations (μM) of the drug undergoing metabolism at 0 and *t* min, respectively. AUC_0-t_ is the change in drug concentration against the time curve from 0 to *t*, *V* is the volume of the incubation solution (μL), SF is a scaling factor set as 135 × 10^6^ hepatocytes/g of liver and 50 g liver/kg body weight in the calculation of the in vitro scaled clearance rate (CL_in vitro_, mL/min/kg), and *N* is normalized to 10^6^ hepatocytes [26]. The predicted hepatic clearance rates were estimated from CL_in vitro_ using the well-stirred model as previously reported [27].

### 2.8. Statistical Analysis

All the experiments were performed at least in triplicate, and their average values with standard deviations were used for statistical analysis. A *p* value of 0.05 or lower was considered to indicate a significant difference.

## 3. Results and Discussion

### 3.1. Microfluidic Device Assembly

**Figure 1:**
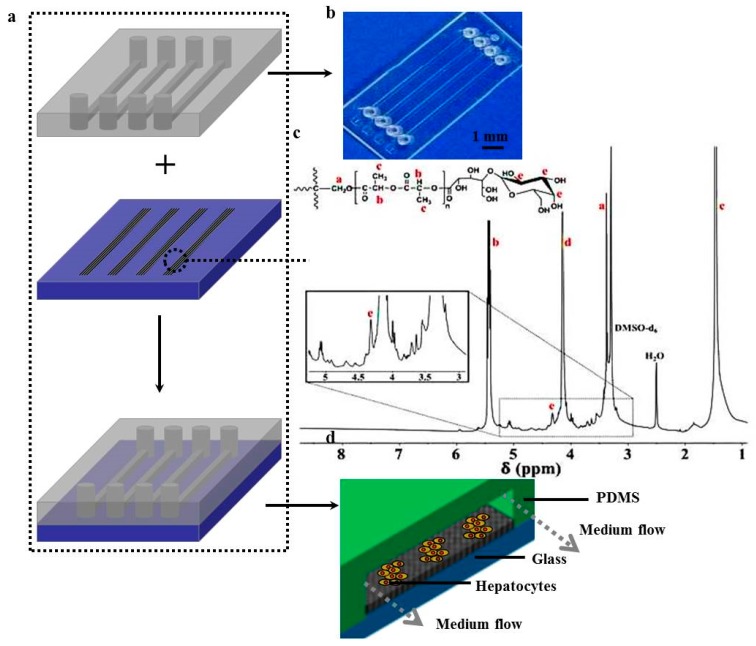
(**a**) Schematic illustration of a microfluidic device assembly; (**b**) digital image of the fiber-based microfluidic chip; (**c**) ^1^H-NMR spectrum of lactosylated poly(dl-lactide) (lac-PLA); (**d**) 3D schematic illustration of the microfluidic device.

Figure 1 shows the schematic diagram of the process of patterning fibers to integrate with a microfluidic chip. The microfluidic chip consists of an upper PDMS channel, middle lac-PLA/PELA fibrous mats, and bottom glass (Figure 1a). Figure 1b shows an image of the PDMS channel, which includes 4 arrayed channels (10 mm long, 200 μm broad); the space between parallel channels is 3 mm. Figure 1c shows the ^1^H-NMR spectrum of lac-PLA. The presence of lactobionic acid and pentaerythritol on the chemical backbone was verified by identifying multiple characteristic peaks at 4 ppm and 3.4 ppm, respectively [17]. The methyl and methane groups of the PLA unit had proton resonances at 1.5 and 5.2 ppm, respectively. The Mn of the polymer estimated from the ^1^H-NMR spectrum corresponded with the feed ratio and GPC analysis, showing that lac-PLA had a *M*_w_ of 7.6 kDa and a *M*_w_/*M*_n_ of 1.32. For the establishment of optimal scaffolds for hepatocyte spheroid formation, electrospun fibers containing PELA and lac-PLA with weight ratios of 1:1 were constructed. Hepatocytes containing asialoglycoprotein receptors (ASGPRs) on the surface can selectively adhere to galactose ligands, inducing the formation of hepatocyte aggregates and a higher level of liver-specific functions. The lac-PLA/PELA fibrous mats used for hepatocyte cultures have uniform pores for efficient mass transfer and are conjugated with galactose ligands for interaction with ASGPR on hepatocytes to enhance hepatocyte functions. The topography of the patterned fibrous mats resembled that of the microchannels, and the thickness of the fibrous mat was approximately 100 μm, which did not block the flow (Figure 1d). The lac-PLA/PELA fibrous mats were placed in the wells of the middle layer at a defined distance from the fluid flow channel to minimize shear stress on the hepatocytes for drug testing. Our previous study also showed that highly porous fibrous mats containing lac-PLA and PELA at a 1:1 ratio with specific ligands could reconstruct the polarity of hepatocytes and achieve prolonged albumin secretion and urea synthesis and higher enzyme activities than those on TCPs, which is important in the establishment of in vitro drug-screening models [28].

### 3.2. Optimization of the Flow-Based Chip

**Figure 2:**
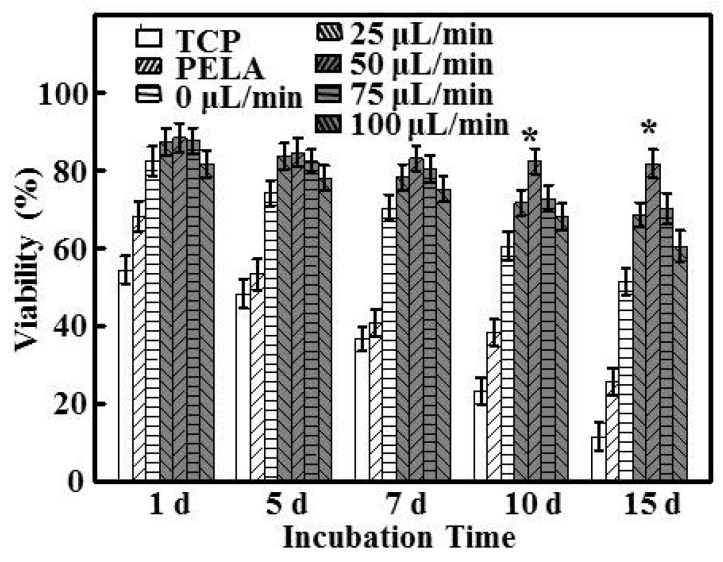
The viability of hepatocytes cultured under different flow rates for 1, 5, 7, 10, and 15 days (*n* = 5, * *p* < 0.05) compared with those cultured on Poly(ethylene glycol)-poly(dl-lactide) (PELA) and tissue culture plates (TCPs).

Hepatocyte culture using the flow-based chip was optimized, and the viability of the hepatocytes cultured under different flow rates (0, 25, 50, 75, 100 μL/min) was compared before culture and 15 days after culture, and among different substrata (Figure 2). There was no significant difference between TCPs and PELA fibrous mats (*p* > 0.05). Hepatocytes on PELA fibrous mats and TCPs were continuously maintained throughout the culture period, leading to a quick decline in hepatocyte viability because such substrata were hindered by diffusion limitations and lacked a nutrient delivery and exchange network.

The number of hepatocytes under a flow rate of 0 μL/min on the chip was significantly higher than that on TCPs or PELA mats after incubation for 15 days (*p* < 0.05), which might be attributed to the lac-PLA content in the blended fibers, which maintains cell viability. Hepatocytes subjected to low flow rates (0 or 25 μL/min) demonstrated no significant effect on cell viability (*p* > 0.05). Cell viability increased significantly with high perfusion rates (50, 75, and 100 μL/min) compared to the low-flow conditions. The cell viability was well retained after incubation for 15 days at a flow rate of 50 μL/min, which was higher than the viability with other flow rates throughout the culture period. A flow rate of 50 μL/min might protect hepatocytes from excessive shear forces of high-flow conditions. At a high-flow rate of 75 μL/min, the spheroids might be flushed out, and therefore, hepatocytes perfused at 100 μL/min showed the lowest viability among the four flow conditions. Hepatocytes cannot settle and form sufficient attachment on the surface under continuous high flow. It is therefore very important to allow the cells to adhere adequately to the substrate before exposing them to continuous low flow. Increasing the perfusion flow rate enhances the delivery of nutrients and removal of waste, but too high a flow rate may induce excessive wall shear stress, which is detrimental to the hepatocytes [29]. Tanaka et al. also demonstrated that the viability of hepatocyte cultures under high shear conditions is lower than that for cultures under 2D conditions [30].

### 3.3. Fluorescence Staining, Size Distribution, and Upscaling the Device

**Figure 3:**
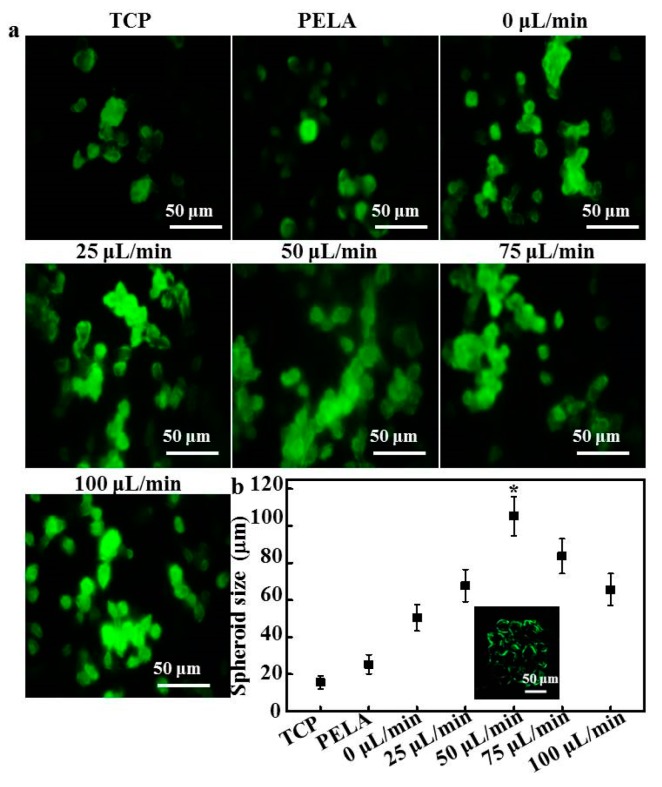
(**a**) Confocal laser scanning microscopy (CLSM) images of immunofluorescent staining of albumin by hepatocytes after 15 days; (**b**) The size distribution of hepatocyte spheroids after 15 days (*n* = 5, * *p* < 0.05); Inset shows a CLSM image of the biliary excretory function of hepatocyte spheroids under a flow rate of 50 μL/min.

Figure 3 shows the fluorescence images and size distributions of the spheroids formed in the microfluidic device. As shown in Figure 3a, rat hepatocytes cultured on TCPs and PELA showed a flat spread pattern and scattered morphology, probably due to the rapid loss of functions of isolated hepatocytes. The efficiency of spheroid formation by hepatocytes cultured under low flow (0 or 25 L/min) was not high during the culture period. Although low flow facilitates spheroids to have close hepatocyte–hepatocyte contact, larger hepatocyte spheroids were not formed. The morphology of hepatocyte spheroids under high flow showed that contact guidance of hepatocytes plays a pivotal role in spheroid formation. The boundaries of hepatocytes became defined, and the spheroids appeared compact after 15 days of culture. Hepatocyte spheroids formed at 50 μL/min flow and were larger than those formed under any other flow rate. Spheroids formed under flow rates of 75 and 100 μL/min were constrained by excessive wall shear stress, which impeded the formation of large hepatocyte spheroids.

Furthermore, the histogram shows that the spheroids formed in the device had a more uniform size distribution (Figure 3a). Figure 3b summarizes the sizes of hepatocyte spheroids cultured on different substrata. The average diameters of hepatocyte spheroids at flow rates of 0 and 25 µL/min were 50.4 ± 7.1 and 67.7 ± 8.2 μm, respectively. Most of the hepatocytes were incorporated into spheroids, with an average diameter of 105.3 ± 11.5 µm after 15 days of culture at 50 µL/min. After culture at flow rates of 75 and 100 µL/min, significantly fewer hepatocytes formed spheroids (average size of 60–80 μm) than those under 50 µL/min flow. In addition, strong fluorescence signals were detected along the border of hepatocytes under 50 µL/min flow, indicating that these hepatocytes maintained their ability to uptake chemicals and efflux bile acid.

Oxygen diffusion has been shown to not be rate limiting, and central hepatocytes did not become hypoxic when the aggregate diameter was of the order of 100 µm or less. Nugraha et al. reported that the combination of physical and chemical cues in a device determine the size of the hepatocyte spheroids. Galactose provides chemical cues to hepatocytes to reorganize into 3D spheroids because the galactose ligand only interacts weakly with ASGPRs in the hepatocyte cell membrane, while the microfluidic structure constrains them and the flow tethers them physically [16].

### 3.4. Hepatocyte Functions

**Figure 4:**
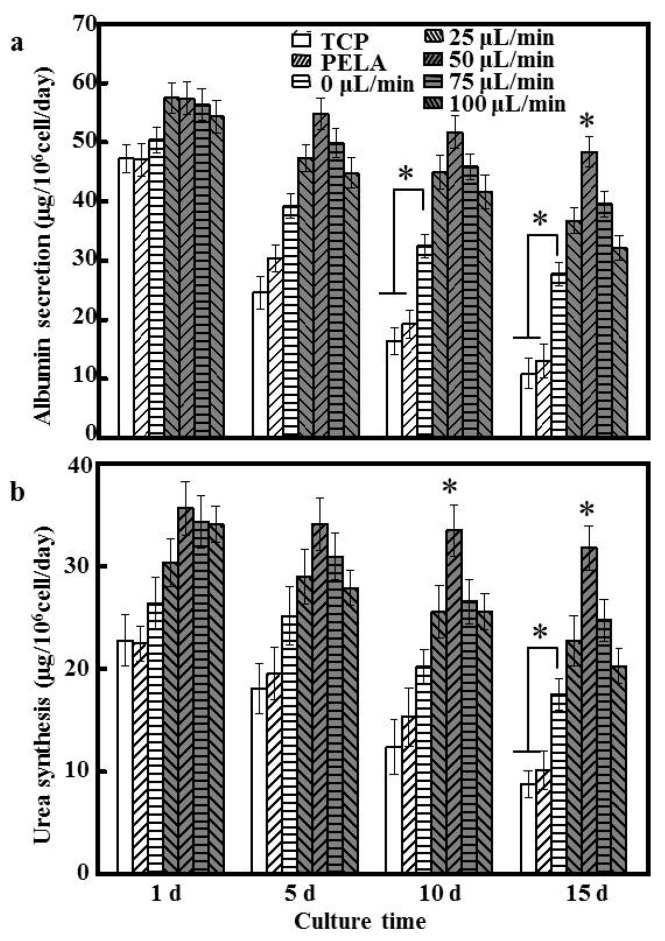
(**a**) Albumin secretion; (**b**) urea synthesis of hepatocytes cultured under different flow rates and substrata over 15 days (*n* = 5, * *p* < 0.05).

To evaluate hepatocyte function, albumin secretion and urea synthesis were measured. We chose these markers because albumin secretion is a specific marker of hepatocyte synthetic function, and urea synthesis is an indicator of xenobiotic metabolism, both of which are important for drug screening. Figure 4a shows the amount of albumin secretion by hepatocytes cultured under different flow rates. These functions were generally better maintained in hepatocytes cultured using the microfluidic device compared to TCPs and PELA mats, at least for 15 days. The albumin secretion of hepatocytes decreased by over 70% for 15 days on PELA fibers and TCPs, indicating that hepatocytes require a dynamic environment for the constant perfusion of cell-culture medium.

A significantly higher amount of albumin secretion occurred under low flow (0 or 25 µL/min) (27.74 ± 1.94 to 36.71 ± 2.05 μg/10^6^ cells/day; *p* < 0.05) for 15 days of culture. Hepatocytes cultured at 50 µL/min showed relatively stable albumin secretion rates, at about 48.37 ± 2.52 μg/10^6^ cells/day after 15 days of incubation. Albumin secretion levels at flow rates of 75 and 100 µL/min were lower than that at a flow rate of 50 μL/min, similar to the trends of viability and spheroid formation of the cultured hepatocytes. Under a flow of more than 25 μL/min, albumin secretion decreased by about 50–10% relative to the other groups. Similar results were noted for urea synthesis. Urea synthesis in hepatocytes cultured under a flow rate of 50 μL/min showed a relatively stable decrease from day 1 (35.61 ± 2.59 μg/10^6^ cells/day) to day 15 of culture (31.77 ± 2.14 μg/10^6^ cells/day), and exhibited the greatest degrees of albumin secretion and urea synthesis throughout the culture period, about 5- and 4-fold higher, respectively, than that of hepatocytes cultured on TCPs for 15 days (*p* < 0.05). Hepatocytes cultured under a flow rate of 50 μL/min maintained hepatocyte functions at a high level for a prolonged period, which is critical in repeated drug exposure and chronic toxicity studies. The differences in the profiles of albumin secretion and urea synthesis might have resulted from their different sensitivities to cell polarity and cell–cell interactions, which depend on different viabilities and functions of hepatocytes [31]. Therefore, 50 µL/min was chosen as the optimal flow rate for subsequent experiments.

### 3.5. Gene Expression of Metabolic Enzymes

**Figure 5:**
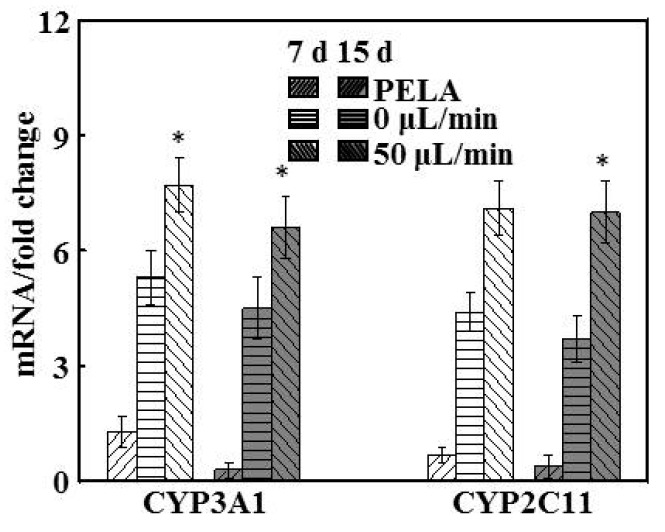
The mRNA levels of CYP3A1, and CYP2C11, normalized to that of β-actin, of hepatocytes for 7 and 15 days (*n* = 5, * *p* < 0.05).

In the rat liver, CYP3A1 is the main CYP3A, and CYP2C11 is its predominant isoform, with both comprising up to 50% of the total CYP content [32]. The forward and reverse primer sequences are listed in Table 1. The maintenance of the activity of the drug-metabolizing enzymes CYP3A1 and CYP2C11 were examined in hepatocytes at flow rates of 0 and 50 μL/min and in PELA mats (Figure 5). At 0 μL/min, hepatocytes showed higher expression levels of CYP3A1 (5.3-fold higher) and CYP2C11 (4.7-fold higher) than those of hepatocytes cultured in PELA. However, there were significant reductions, by around 10.2% and 8.4%, respectively, in the mRNA levels of CYP3A1 and CYP2C11 enzymes at 0 μL/min for 15 days, compared with those after 7 days. Hepatocytes at 50 μL/min could retain significant higher expression levels of liver-specific genes relevant for the enzymes’ activity and drug metabolism, and the results are in accord with the function of those hepatocytes.

### 3.6. Hepatocyte Drug-Metabolizing Enzymes

**Figure 6:**
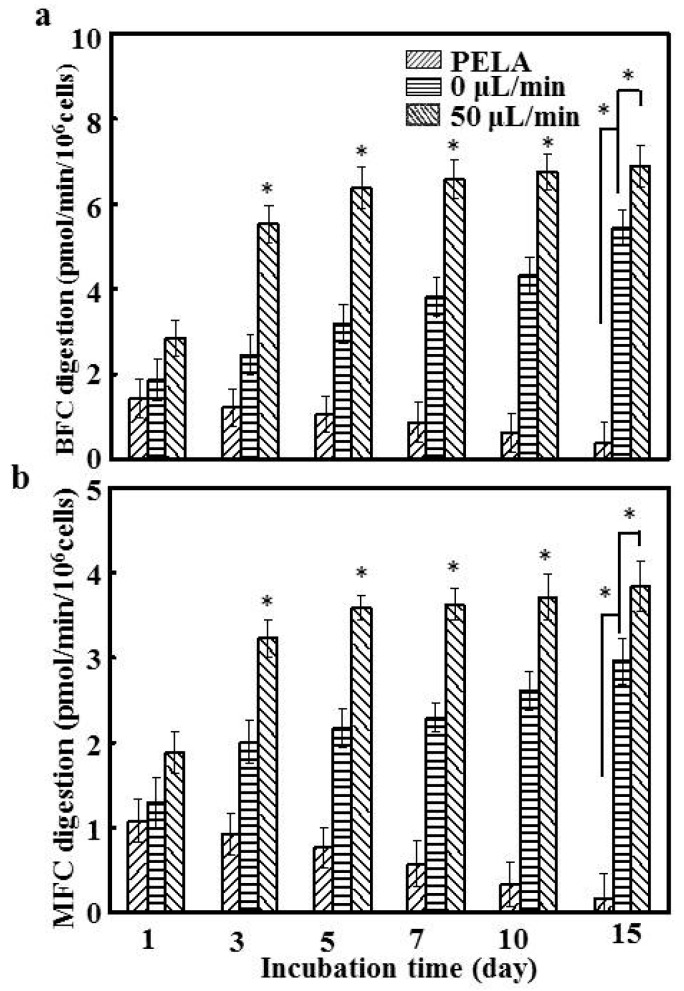
The activities of (**a**) CYP3A1 and (**b**) CYP2C11 enzymes were detected by the specific fluorescent substrates 7-Benzyloxy-4-trifluoromethylcoumarin (BFC) and 7-methoxy-4-trifluoromethylcoumarin (MFC), respectively (*n* = 5, * *p* < 0.05).

Given that BFC and MFC are the specific substrates for CYP3A1 and CYP2C11, we investigated the effect of drug-metabolizing enzymes throughout the culture period [33]. The temporal trend in metabolite formation rates paralleled that of mRNA expression; hepatocytes on PELA fibrous mats demonstrated a rapid decrease in the enzyme activities. CYP3A1 and CYP2C11 enzymatic activities in PELA fibrous mats were much lower and fluctuating than hepatocytes on the chip at a flow rate of 0 μL/min (*p* < 0.05). This can be ascribed to the nutrient diffusion limitations affecting cellular viability [34], resulting in lower BFC and MFC formation (Figure 6). lac-PLA enhanced hepatocyte function, and there was a continuous increase in the activities of CYP3A1 and CYP2C11 enzymes in hepatocytes at 0 μL/min during the 15-day incubation. Hepatocytes at a flow rate of 50 μL/min were therefore comparatively more stable and maintained the highest enzyme activities among the groups, reaching 6.7 and 3.8 pmol/min/10^6^ cells for CYP3A1 and CYP2C11, respectively. These data suggest that the dynamic culture system with continuous closed recycling of the medium is necessary for hepatocytes that mimic the in vivo environment [35]. The level of activity of CYP3A1 and CYP2C11 enzymes showed a similar trend until 15 days (Figure 5). After culture for 15 days, the CYP3A1 and CYP2C11 activities of the hepatocytes at a flow of 50 μL/min were more than 3.9- and 3.4-fold higher, respectively, than those of hepatocytes cultured on PELA. This implies that basal CYP450 activity is maintained for longer periods, which is important for minimizing drug screening variation caused by functional fluctuation or deterioration [36]. Thus, in hepatocytes subjected to a flow rate of 50 μL/min, the retention, synthetic functions, and enzyme activities in the presence of both galactose and flow were superior to or comparable with other conditions.

### 3.7. Drug Metabolism Tests on Cultured Hepatocytes

**Figure 7:**
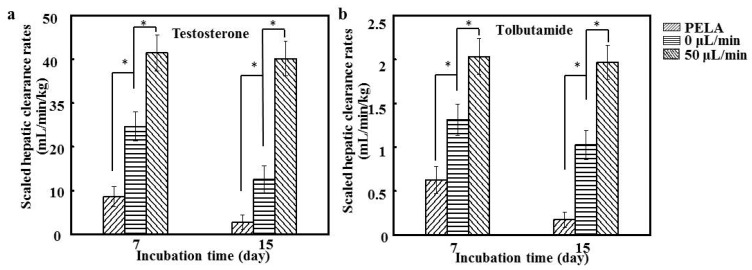
(**a**) The in vitro scaled clearance rates of tolbutamide and (**b**) testosterone during 2 h of metabolism by hepatocytes after 7 and 15 days (*n* = 5, * *p* < 0.05).

To clarify the prediction ability of hepatocytes in the drug metabolism screening, the concentration changes in testosterone and tolbutamide during 2 h of metabolism by hepatocytes were recorded, and the predicted clearance rates were calculated for 7 and 15 days. Tolbutamide and testosterone were used as the substrates of hepatocyte CYP3A1 and CYP2C11 enzymes, respectively, for the drug metabolism study [37]. Figure 7a shows that the clearance rates of tolbutamide by hepatocytes at a flow of 0 μL/min were significantly higher than those of hepatocytes cultured on PELA fibrous mats (*p* < 0.05), being around 45.4% higher after 7 days. The results of testosterone metabolism by CYP2C11 indicated a profile similar to that of CYP3A1 (Figure 7b). The clearance rates of both substrates appeared to be the highest for hepatocytes at a flow of 50 μL/min (*p* < 0.05). Although there was a slight decrease in the drug clearance rate for hepatocytes after the 15-day culture, the enhanced metabolic capabilities were retained for hepatocytes at a flow rate of 50 μL/min. The scaled clearance rates of tolbutamide and testosterone for hepatocytes at a flow of 50 μL/min (1.98 ± 0.43 and 40.80 ± 10.13 mL/min/kg) were around 2.1- and 2.5-fold higher, respectively, than those at a flow of 0 μL/min. In addition, these drug clearance rates were comparable to the in vivo values in rats (2.46 mL/min/kg for tolbutamide and 45.1 mL/min/kg for testosterone) [37]. In an earlier study, we found that hepatocytes cocultured with other cells exhibited an excellent ability for drug metabolism, but other types of cells might require inconvenient and complicated procedures [37]. We therefore developed patterned fiber-embedded microfluidic chips, which could produce sensitive and consistent responses to nano-Ag-induced hepatotoxicity, but primary hepatocytes only adhering to the surface of PLA electrospun mats have certain limitations [20]. Previous reports have shown that the interaction between galactose ligands and ASGPR (on the surface of hepatocytes) induces the formation of hepatocyte aggregates, and hepatocytes can specifically adhere to surfaces conjugated with galactose ligands [17]. In this study, hepatocytes seeded on lac-PLA under a microfluidic culture were compared with those of other culture/coculture methods. Our findings highlight the correspondence between in vitro and in vivo conditions in rats. A flow rate of 50 μL/min for hepatocytes not only provides good predictability for hepatic clearance but is also advantageous in maintaining metabolic competency for longer periods of time [38]. Thus, fiber-embedded PDMS chips for hepatocyte culture have promising potential as an in vitro model to predict drug metabolism in vivo.

## 4. Conclusions

We report a fiber-embedded PDMS chip for uniformly sized hepatocyte spheroid formation and culture. Compared with other hepatocyte cultures, hepatocytes at a flow of 50 μL/min demonstrated significantly higher levels of enzyme activities and gene expression, better predicted drug clearance, and better in vitro–in vivo correspondence. The microfluidic device provides a useful platform to construct liver tissue in vitro, and it can serve as an in vitro testing model for the evaluation of drug metabolism.

## Figures and Tables

**Table 1:** Primer sequences for real-time PCR.

Gene Name	Sequence
CYP3A1	Forward:5’-TATGGGGAAAGCCATCTCTG-3’ Reverse: 5’-CAGGTTTGCCTTTCTCTTGC-3’
CYP2C11	Forward:5’-AGGGCCTTGGAGTCATTTTT-3’ Reverse:5’-GCACCTTTGCTCTTCCTCAG-3’
β-actin	Forward:5’-ACCCCAAAGCCAACAGAGAG-3’ Reverse:5’-AGGCATACAGGGACAGCACA-3’
